# Small-Sized Nanophosphorus Has a Positive Impact on the Performance of Fenugreek Plants under Soil-Water Deficit Stress: A Case Study under Field Conditions

**DOI:** 10.3390/biology11010115

**Published:** 2022-01-12

**Authors:** Alaa I. B. Abou-Sreea, Marwa Kamal, Dalia M. El Sowfy, Mostafa M. Rady, Gamal F. Mohamed, Sami A. Al-Dhumri, Mohammad S. AL-Harbi, Nasr M. Abdou

**Affiliations:** 1 Horticulture Department, Faculty of Agriculture, Fayoum University, Fayoum 63514, Egypt; aib00@fayoum.edu.eg; 2 Clinical Pharmacy Department, Faculty of Pharmacy, Fayoum University, Fayoum 63514, Egypt; mka05@fayoum.edu.eg; 3 Soils and Water Department, Faculty of Agriculture, Fayoum University, Fayoum 63514, Egypt; dms00@fayoum.edu.eg (D.M.E.S.); nma02@fayoum.edu.eg (N.M.A.); 4 Botany Department, Faculty of Agriculture, Fayoum University, Fayoum 63514, Egypt; gfm00@fayoum.edu.eg; 5 Department of Biology, Al Khurma University College, Taif University, P.O. Box 11099, Taif 21944, Saudi Arabia; A.sami@tu.edu.sa; 6 Department of Biology, College of Science, Taif University, P.O. Box 11099, Taif 21944, Saudi Arabia; mharbi@tu.edu.sa

**Keywords:** *Trigonella foenum-graecum*, growth and seed yield, irrigation water limitation, nanophosphorus, physio-biochemical indices, trigonelline, anatomy

## Abstract

**Simple Summary:**

Experiments with fenugreek plants have provided insight into tolerance to deficit irrigation (dI) stress, the way in which fenugreek alters its morpho-physio-biochemical and anatomical responses when nanophosphorus (nP) is administered against dI stress. Foliar nourishing with nP effectively improved biomass, productivity, water use efficiency (WUE), photosynthetic pigments, leaf tissue integrity, and anatomical features in the dI-stressed fenugreek plants. Therefore, nP reduced the negative effects of dI and increased dI stress tolerance, plant growth and productivity by increasing WUE, osmo-regulatory compounds (especially, soluble sugars and proline) and trigonelline, along with the antioxidant (ascorbate, glutathione, phenolics, and flavonoids) activity, which serve as potent defenses to protect plants from dI stress.

**Abstract:**

Phosphorus (P) is an essential macronutrient necessary for plant growth, development, and reproduction. Two field experiments were carried out in 2018/2019 and 2019/2020 on P-deficient soil to evaluate the impact of foliar fertilization with nanophosphorus (nP) on growth, yield, and physio-biochemical indices, as well as trigonelline content of fenugreek plants under deficient irrigation (dI) stress (a deficit of 20 and 40% of crop evapotranspiration; dI-20 and dI-40). The growth and yield traits, leaf integrity (relative water content and membrane stability index), photosynthetic pigment contents, leaf and seed P contents, and stem and leaf anatomical features significantly decreased under dI-20, with greater reductions recorded under dI-40. In contrast, water-use efficiency, osmoprotective compounds, including free amino acids, soluble sugars, proline, and trigonelline, along with antioxidant contents (ascorbate, glutathione, phenolics, and flavonoids) and their activity increased significantly under both dI-20 and dI-40. However, foliar feeding with nano-P considerably increased plant growth and yield traits, leaf integrity, photosynthetic pigments contents, leaf and seed P contents, and anatomical features. Besides, water-use efficiency, osmoprotectant contents, and antioxidant content and activity were further increased under both dI-20 and dI-40. The positive effects were more pronounced with the smaller nP (25 nm) than the larger nP (50 nm). The results of this study backed up the idea of using foliar nourishment with nP, which can be effective in modulating fenugreek plant growth and seed production.

## 1. Introduction

Fenugreek (*Trigonella foenum-graecum*) is a self-pollinating annual leguminous crop belonging to the family *Fabaceae*. It is widely cultivated in Mediterranean countries and Asia for its seeds, which possess key medicinal properties [1,2]. It is one of the most promising medicinal herbs known since antiquity and has great nutritional value as well. The seeds contain several important secondary metabolites that have beneficial medicinal roles, including trigonelline. Trigonelline plays a useful role in overcoming the pathological symptoms of Alzheimer’s disease. It is also a neuroprotective, antidepressant and anti-anxiety agent. Besides, it has a modulating effect on cognitive functions and Parkinson’s disease [3]. In most countries, including Egypt, India, China, and some parts of Europe, the seeds have been used for their beneficial health effects of being galactagogue, anti-inflammatory, anti-cancer, antioxidant, anti-microbial, anti-ulcer, anti-obesity, anti-hyperglycemic, anti-cholesterol, etc. [4,5,6,7,8]. Trigonelline is a pyridine alkaloid, first isolated from dry fenugreek seeds that are rich in this medicinal compound [9,10]. It has different physiological activities in plants, affecting the plant cell cycle [10,11]. It is a necessary nutritional source for the symbiotic bacteria of nodulating plants [12]. In response to stress conditions, trigonelline accumulates to function as an osmo-regulatory compound [10,13]. During early seedling establishment, it migrates from seed cotyledons to other plant tissues to exert its physiological impact [14].

Fenugreek, like any other crop, is negatively affected throughout its life cycle upon exposure to different environmental stresses [15,16,17]. The stresses affecting different crops include salinity [18,19,20,21], cadmium stress [22], nutrient deficiency [23], high carbonate content [24,25], and drought [26]. Availability of irrigation water is the most limiting factor for agricultural sustainability and food security worldwide, especially in dry regions, including Egypt. In these regions, deficit irrigation water (dI) is the main limiting factor that threatens crop productivity. The gap between water demand and supply is about 13.6 billion cubic meters (BCM) per year. Agriculture requires more than 80% of available water resources. Minimizing water inputs and maximizing crop productivity by adopting more efficient irrigation scheduling strategies is of vital importance and a major challenge under dI conditions [21,22,23,24,25,26,27].

Stress of dI causes metabolic, biochemical, and physiological changes in plants. It causes negative impacts on growth and yield, plant water status and water relations, membrane integrity and stability, pigment content, and photosynthetic activity. It also negatively affects plant phosphorus (P) content and anatomical features [21,22,23,24,25,26]. However, the water use efficiency (iWUE) improves under dI stress [26]. Other positive changes under dI stress include osmo-regulatory adaptation and the accumulation of antioxidants and trigonelline [15,16,17,18,19,20,21,22,23,28,29,30]. Fenugreek plant growth and productivity decrease dramatically with increasing dI [31]. Significant losses in crop productivity under dI stress have been reported mainly due to abnormal performance of all traits related to photosynthesis, especially leaf pigments and their activities [26,28].

As reported in [32], not all of the conventional fertilizer (cFs) dosage particles used can reach the plant due to many factors like leaching/infiltration, run-off, evaporation, hydrolysis by soil moisture, and degradation by soil flora. Besides, the solubility of various chemical compounds and macromolecules of certain nutrients, like P, make them not easily available for uptake by plant roots due to their interaction with soil ingredients [e.g., minerals (inorganic) or humic and fulvic acids (organic)]. As mentioned in [33], about 40–70% of N, 80–90% of P, and 50–90% of K fertilizers are lost within soil and do not reach the plants. Therefore, applying fertilizers to soils to compensate for their losses will negatively affect the nutrient balance [34]. Besides, excessive amounts of mineral fertilizers such as N or P sources have negative impacts on both soil and groundwater due to leaching of residual minerals, which negatively affects both sustainability and crop yield [35]. To overcome such a problem, researchers are trying to develop an efficient and eco-friendly production technology based on innovative techniques to achieve high plant performance via sustainable treatments [36].

As an alternative solution to promote food production without any negative influence on the ecosystem, nanotechnology has emerged and has been applied in the field of agriculture. Nanofertilizers (NFs) are synthesized from cFs, or extracted from various vegetative or reproductive parts of the plant using different chemical, physical, mechanical, and biological methods [37]. In this technology, nutrients can be encapsulated with nanomaterials and coated with a protective film, or delivered as emulsions or nanoparticles (NPs) [38]. These active ingredients (NPs) have a diameter of 1–100 nm and have a large specific surface area that can lead to an acceptable reactivity that in turn elevates the effective absorption of nutrients and components essential for plant growth and metabolism [39]. The NPs possess tremendous physicochemical properties such as smaller size, high area to volume, enhanced reactivity, hyper ionizing strength, improved chemical stability, higher absorbability, increased tolerance to pH, and expanded thermal stability. Therefore, NPs are environmentally friendly and have the potential to increase soil fertility, improve yields, reduce pollution, and increase microbial activities [40]. As one of the advantages of NFs, they can be used as a foliar spray in smaller quantities compared to cFs. This strategy can minimize fertilizer losses and application cost, and maximize plant productivity through effective management [19,24,25,41,42]. Compared with most cFs, NFs are more effective due to their nature, which can permit slow release and enhance efficient nutrient uptake by absorbing roots [43]. Therefore, this strategy could provide a platform for sustainable nutrient (P) delivery systems that exploit the nanoporous surfaces of plant parts on its surfaces [44]. Depending on their solubility and their transport pathways, NPs are sometimes modified and coated with compounds such as chitosan, citric acid, polyacrylic acid, zeolite, montmorillonite, or bentonite nanoclays [45].

The use of nanophosphorus (nP) has remarkable enhancing effects on plant growth and production by improving physio-biochemical indices and antioxidant defense strategies as a result of relieving abiotic stress. Foliar nourishment with nP noticeably improves growth, yields, leaf chlorophyll, photosynthetic efficiency, membrane stability index, relative water content, various antioxidant activities, P content, and various osmo-regulatory compounds in *Phaseolus vulgaris* and *Triticum aestivum* plants [24,46]. Since P is an essential component of amino acids, high-energy molecules (ADP and ATP), nucleic acids, and phospholipids in plants [47], it is, therefore, a pivotal nutrient essential for plant growth, development, and reproduction. This study aimed to evaluate the effect of nP on morpho-physio-biochemical indices of fenugreek plants grown under two levels of deficit irrigation (dI) stress using P-deficient soil. The antioxidant activity, anatomical features, yield parameters, and irrigation water-use efficiency were also evaluated. As this study is the first to use nP at two nanoscales versus conventional P (cP) for dI-stressed fenugreek plants, it was hypothesized that smaller nP would outperform larger nP scale, which would, in turn, outperform cP in improving all of the tested measurements of dI-stressed plants. Trigonelline (a medicinal alkaloid compound) was also investigated for the expected improvement in fenugreek seeds produced under dI stress conditions.

## 2. Materials and Methods

### 2.1. Experimental Soil Location, Properties, Meteorological Characteristics, Layout, Treatments, and Nanoparticle Preparations

An experimental clayey soil with fair physical and chemical properties (Table 1; [48]) was used for two experiments during the 2018/2019 and 2019/2020 seasons. It is located in the experimental farm (29°17′ N; 30°53′ E) of the College of Agriculture, Fayoum University, Egypt.

**Figure 1:**
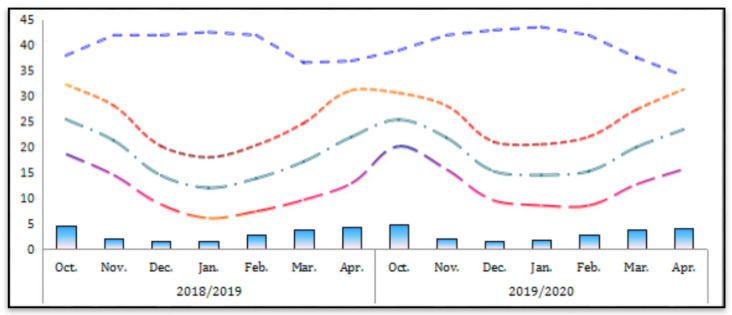
Monthly meteorological parameters in Fayoum Governorate from October to April during the 2018/2019 and 2019/2020 seasons.

The local weather characteristics of the location were obtained from the Fayoum Meteorological Station (Figure 1). The average maximum and minimum temperatures remained at 31.45 and 7.25 °C in October and January, respectively. The relative humidity ranged between 36.6 and 43.6%. E-pan evaporation rates coincided with air temperatures with the highest and lowest mean evaporation rates of 4.65 and 1.55 mm day^−1^ in October and December, respectively.

The experiments were conducted in a complete randomized design using a split-plot arrangement with three replications. Irrigation treatments were applied at three levels, including full watering (dI-00, control), deficit irrigation at 20% of crop evapotranspiration (ETc) (dI-20), and watering reduction at 40% of ETc (dI-40). The three irrigation treatments were imposed on the main plots of the experimental soil. For each of the three irrigation treatments, phosphorus (P) was applied for three treatments, namely conventional phosphorus (cP), phosphorus nanoparticles of 50 nm (nP-1), and phosphorus nanoparticles of 25 nm (nP-2). The three fertilization treatments were imposed on the subplots of the experimental soil. Both the irrigation and fertilization treatments consisted of nine combination treatments with three replications each, giving a total of 27 plots. The area of each experimental plot was 6 m^2^ (2.5 m × 2.4 m) with four rows of 2.5 m in length and 60 cm apart.

Fertilization with cP was applied to the plots before sowing at 400 kg P_2_O_5_ ha^−1^ (0.24 kg plot^−1^) sourced from calcium superphosphate (15.5% P_2_O_5_). Using the foliar spray technique, nP-1 and nP-2 were sprayed twice on days 30 and 60 from sowing. Each nP (prepared from calcium phosphate, 18% P and 6% Ca) was sprayed at a concentration of 0.1 g L^−1^ using a 20 L dorsal Sprayer (model 0417.02.00; Guarany Ind. & Com. Ltd., Itu, Sao Paulo, Brazil). A few drops of Tween-20 were added to the spraying solutions as a surfactant. The solutions were sprayed onto the upper leaf surface in the early morning before sunrise. Full irrigation was given for all treatments until full emergence of seedlings, which was observed two weeks after sowing. Then, dI-00, dI-20, and dI-40 treatments were applied.

**Figure 2:**
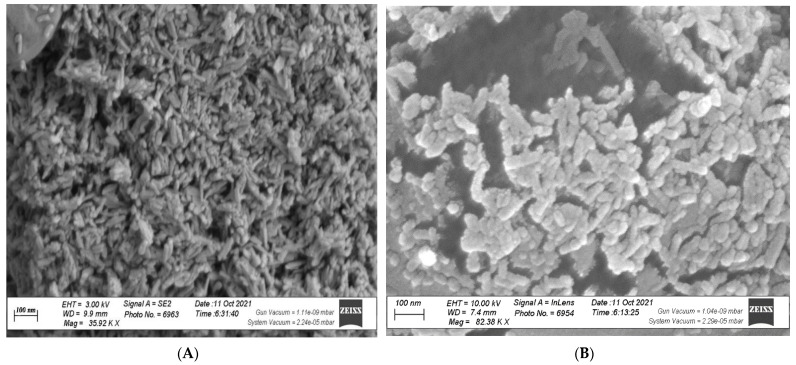
TEM image of zinc oxide nanoparticles (ZnONPs). (**A**) (25 nm), (**B**) (50 nm).

The technique of Eleyan et al. [49] was utilized to prepare nP in the Laboratory using ball milling (Photon Company, Cairo, Egypt). Transmission Electron Microscopy (TEM) was utilized to evaluate and measure the size of nP particles (50 nm and 25 nm) utilizing JEOL transmission electron microscope (JEM-1400 TEM, Tokyo, Japan) with the technique of Wang et al. [50]. The prepared P in two nanoscales (25 and 50 nm) are shown in Figure 2.

### 2.2. Irrigation Treatments and Water Applied

For applying irrigation treatments, the values of crop water consumption (crop evapotranspiration; ETc) were computed by the following Allen et al. [51] formula:

ETc = Epan × Kpan × Kc(1)

where ETc = consumption (mm d^−1^) of water by crop, Epan = evaporation (mm d^−1^) from the Class A pan, Kpan = coefficient of pan evaporation, and Kc = coefficient of crop.

In addition, the following equation was also used to compute irrigation water applied (IWA) [51]:

IWA = (A × ETc × Li × Kr)/(Ea × 1000)(2)

where IWA = irrigation requirements (m^3^), A = the area (m^2^), ET_c_ = crop water evapotranspiration (mm d^−1^), Li = irrigation intervals (day), Kr = covering factor, and Ea = efficiency (%) of application. IWA for each irrigation treatment is shown in Table 2.

The IWA of each experimental plot was controlled and conveyed utilizing a “1” plastic pipe (spile) with a diameter of 5 cm per plot, and the time of irrigation was adapted according to the spile discharge calculated by Israelsen and Hansen [52].

(3) $$Q=CA\sqrt{2gh}\times 0.001$$

where *Q* = plastic pipe discharge (l s^−1^), *C* = discharge coefficient, *A* = pipe cross section area (cm^2^), *g* = acceleration of gravity (cm s^−2^), and *h* = average of an effective head of water (cm).

### 2.3. Field Agro-Management Practices

Before sowing, the field was disked and harrowed. The seeds were sown by a thread in the open field in the third week of October during both the 2018/2019 and 2019/2020 seasons. The seeds were sown by hand and irrigated immediately. Sowing was done at a depth of 0.5−1 cm in rows with a seeding level of 100 kg ha^−1^ (60 g seeds per plot; 6 m^2^). The fenugreek seeds were obtained from the Department of Medicinal and Aromatic Plants, Ministry of Agriculture, Giza, Egypt. Organic manure was added at 25 m^3^ ha^−1^ as a basic dose to all experiments. Nitrogen fertilizer was added at 40 kg N ha^−1^ sourced from ammonium sulfate (20.5% N) and potassium fertilizer was added at 35 kg K_2_O ha^−1^ sourced from potassium sulfate (48% K_2_O). They were added in three equal doses to the plots on days 35, 56, and 77 of sowing. All other farming practices necessary for fenugreek growth, development, and commercial production were identically operated following the recommendations of the Egyptian Ministry of Agriculture.

### 2.4. Morphological and Yield Characteristics

On day 120 from sowing (at full blooming; the third week of February in both seasons), nine plants were randomly chosen from each treatment (three plants from each plot) and harvested to determine morphological characteristics, namely plant height (cm), root length plant^−1^ (cm), and dry weight plant^−1^ (g).

On day 190 from sowing (at fruiting stage), nine samples were randomly taken for morpho-physical, biochemical, and anatomical traits. All remaining plants in all plots were subjected to estimation of yield traits, namely mean number of pods plant^−1^, mean seed yield plant^−1^ (g), and the total seed yield was calculated as ton ha^−1^.

### 2.5. Irrigation Water Use Efficiency (iWUE)

Jensen’s method and equation [53] were used for calculating iWUE values, which were computed as kg (yield) m^−^^3^ of water for the treatments after harvesting. The following formula was applied:

iWUE = [seed yield (kg ha^−^^1^)]/[water applied (m^3^ ha^−^^1^)] (4)

### 2.6. Leaf Integrity

Relative water content (RWC, %) was estimated and computed utilizing Osman and Rady’s method [54]. The following formula was applied:

RWC (%) = [(fresh mass − dry mass)/(turgid mass − dry mass)] × 100(5)

where fresh mass = weight of leaf tissue sample immediately after sampling, turgid mass = weight of the same sample after saturation in double-distilled water in the dark for 24 h, and dry mass = weight of the same sample after drying at 70 °C for 48 h.

Membrane stability index (MSI, %) was estimated using Rady’s method [55]. The following formula was applied:

MSI (%) = [1 − (EC_1_/EC_2_)] × 100 (6)

where EC_1_ = electrical conductivity value in leaf tissue sample solution after heating at 40 °C for 30 min, and EC_2_ = electrical conductivity value in another leaf tissue sample solution after heating at 100 °C for 10 min.

### 2.7. Leaf Pigments, Free Amino Acids (FAa), Soluble Sugars (Ss), and Proline Measurements

Lichtenthaler’s method [56] was used to estimate the total chlorophyll and carotenoid contents in fresh leaf samples collected on day 120 of sowing. Dubey and Rani’s method [57] was applied to extract and quantify FAa. Extraction was done for 0.2 g dried leaf sample utilizing 10 mL of 80% (*v*/*v*) ethanol. Filtration was followed to obtain the extract (0.1 mL). The extract received 5 mL ninhydrin reagent. The mixture was exposed to vigorous shaking, heating in a boiling water bath for 10 min, and cooling. Absorbance values of the mixture were recorded at 570 nm. The method of Irigoyen et al. [58] was utilized to extract and quantify Ss. Extraction was done for 0.2 g dried leaf sample utilizing 5 mL C_2_H_5_OH (96%, *v*/*v*), and then washed with 5 mL C_2_H_5_OH (70%, *v*/*v*). For the resulted extract, centrifugation was practiced at 3500× *g* for 10 min. Before measurement, the resulted supernatant was stored at 4 °C. The Ss content was quantified by reacting C_2_H_5_OH-extract with a fresh reagent (150 mg anthrone plus 100 mL H_2_SO_4_ 72%) utilizing a boiling water bath for 10 min. The mixture was cooled, and then absorbance values were taken at 625 nm. The method of Bates et al. [59] was applied to extract and quantify the total free proline. Extraction was done for a 0.5 g dried leaf sample utilizing sulfosalicylic acid (10 mL, 3%) followed by centrifugation (10,000× *g* for 10 min) for the extract. A 2 mL solution of acid–ninhydrin was added to the supernatant, and the mixture was exposed (an incubation) to 90 °C for 0.5 h. After terminating the reaction, toluene was utilized for another extraction. The toluene phase was utilized to read the absorbance at 520 nm against a standard curve. All these measurements were performed utilizing a Spectrophotometer (Thermo Bausch and Lomb-2000 Spectronic, Mercers Row, Cambridge, UK).

### 2.8. Leaf and Seed Phosphorus (P) Measurements

From each treatment, leaf and seed samples were selected, washed using distilled water, and dried (on 70 °C for 48 h) to estimate leaf and seed contents of P using Inductively Coupled Plasma–Optical Emission Spectrometry (ICP-OES, Perkin-Elmer OPTIMA-2100 DV, Norwalk, CT, USA) apparatus. The P measurements were carried out in the laboratory of the Soil and Water Department, Agriculture College, Fayoum University, Egypt.

### 2.9. Ascorbate (AsA) and Glutathione (GSH) Measurements

The two methods of the two papers [60,61] were practiced for determining leaf contents (expressed as µmol g^−1^ fresh leaves) of AsA and GSH, respectively. For AsA content, a mixture consisted of 30 mM of K-P-buffer (pH 7.4), 2.5% TCA solution, 8.4% H_3_PO_4_ solution, 0.8% bipyridyl solution, and 0.3% FeCl_3_ solution was supported by the leafy extract. The reaction was carried out at 40 °C for 0.5 h. The absorbance was taken at 525 nm. For GSH, a 0.5 g leaf sample was homogenized in a 2% metaphosphoric acid solution. The extract was centrifuged at 17,000× *g* for 10 min. The produced supernatant was received 10% sodium citrate solution for neutralization. A 1.0 mL assay was prepared and stabilized for 3–4 min at 25 °C. Glutathione (GSH) reductase was then added to measure GSH content by taking the absorbance at 412 nm against a standard curve of GSH.

### 2.10. Antioxidative Activity Measurement

The antioxidative activity (AAc) was measured as DPPH assay following the method in Brand-Williams et al. [62]. For the DPPH assay, to prepare a solution of 2,2-Diphenyl-1-Picrylhydrazyl, 3.8 mg was dissolved in MeOH (25 mL). The prepared solution (100 µL) was added, individually, into plate wells (96-well each) with the addition of 85 µL MeOH + 15 µL seed extract. At room temperature in the dark, the plates were incubated for 0.5 h, and then absorbance was read at 515 nm using a microplate reader (iMarkTM, Bio-Rad, Vienna, Austria). The calibration curve was made with Trolox and the AAc was expressed as mg Trolox equivalents g^−1^ seed DW.

### 2.11. Measurements of Trigonelline, Total Phenolics (TPhs), and Total Flavonoids (TFs) Contents

Seed samples were grounded to a fine powder with an electric mill before the measurements of trigonelline, TPhs, and TFs were performed. For trigonelline, a 0.2 g powdered seed sample was sonicated utilizing 10 mL C_2_H_5_OH by an ultrasonic bath for 0.5 h. Then, the mixture was centrifuged (5000 rpm for 10 min) and evaporation was done for the supernatant, followed by dissolving the residue utilizing 2 mL C_2_H_5_OH and then stored at 4 °C. Trigonelline content was evaluated by HPLC (Agilent Technologies, Santa Clara, CA, USA) system, detecting trigonelline absorbance at 263 nm [63]. For TPhs, the method of Folin–Ciocalteu reagent was applied following a procedure of [64]. A mixture of the Folin–Ciocalteu reagent (5 µL) + distilled water (100 µL) + seed sample extract (10 µL) was shaken and then distilled water (125 µL) and Na_2_CO_3_ (10 µL; 35%) were added. At room temperature in the dark, an hour of incubation was done. The absorbance reading was taken at 750 nm. A calibration curve was made utilizing caffeic acid, and the total seed TPhs was expressed as mg caffeic acid (CA) equivalents g^−1^ seed DW. For TFs, the modified method in [65] was applied. A mixture of seed extract (40 µL) + distilled water (100 µL) + AlCl_3_·6H_2_O-solution (15 µL, 10%) + NaNO_2_-solution (15 µL, 2.5%) was prepared. After shaking for 5 min, NaOH (50 µL 1 M) was added, and another 5 min of shaking was done. The solution absorbance was noted at 490 nm. Different rutin concentrations were prepared following the same method as a calibration curve, and the TFs values were expressed as mg rutin (RU) equivalents g^−1^ seed DW.

### 2.12. Anatomical Attributes

On day 45 of sowing, anatomical studies were performed on five (stem and leaf) samples selected randomly from each treatment. Samples were killed and fixed for 48 h in FAA solution. Samples were washed in 50% C_2_H_5_OH, dehydrated, and cleared in tertiary butyl alcohol series, embedded in paraffin wax of 54–56 °C m.p. A rotary microtome was utilized to cut cross-sections at 20 μ thick and adhered to Haupt’s adhesive. Sample staining was done utilizing the crystal violet-erythrosin combination [66]. Carbol-xylene and Canada balsam were utilized for clearing and mounting, respectively. An upright light microscope (AxioPlan, Zeiss, Jena, Germany) was utilized to document the sections. A micrometer eyepiece was used for measurements.

### 2.13. Statistical Analysis

The data were analyzed utilizing ANOVA for a randomized complete blocks design, after testing for homogeneity of error variances [67] using InfoStat software estadistico [68]. Duncan’s Multiple Range Test was practiced at a 5% level of probability to test the differences between treatment means.

## 3. Results

### 3.1. Effect of Irrigation Deficiency and Nanophosphorus on Growth and Yield Traits

Deficit irrigation water (dI) by 20 and 40% of crop evapotranspiration (dI-20 and dI-40, respectively) negatively affected fenugreek plant growth and output performance, but positively affected irrigation water use efficiency (iWUE), as well as leaf nourishing with nanophosphorus (nP) positively affected these traits under normal conditions or dI stress (Table 3 and Table 4).

The dI-20 significantly decreased plant height (PHt), root length (RLh), plant dry weight (PDWt), pods number per plant (PNpP), seed weight per plant (SWpP), and seed yield per hectare (SYpH) on average by 9.8 and 11.0%, 19.6 and 12.0%, 15.2 and 17.7%, 23.2% and 19.4%, 25.6 and 18.7%, and 9.4 and 8.5% in 2018/2019 and 2019/2020, respectively, while iWUE was increased on average by 12.9 and 13.5%, respectively, when compared to the control. The dI-40 further decreased PHt, RLh, PDWt, PNpP, SWpP, and SYpH on average by 20.9 and 22.3%, 33.0 and 35.4%, 34.5 and 34.4%, 45.2 and 48.4%, 40.4 and 43.7%, 32.9 and 31.7% in both growing seasons, respectively, however, the iWUE results obtained under the dI-40 were similar to those obtained under the dI-20, with an increase of 11.4 and 13.5% compared to the control, respectively.

Under all irrigation regimes, leaf nourishment with nP-1 significantly increased PHt, RLh, PDWt, PNpP, SWpP, SYpH, and iWUE on average by 4.7 and 4.6%, 7.5 and 11.7%, 10.6 and 8.5%, 10.4 and 11.0%, 8.3 and 10.1%, 3.9 and 3.8%, and 12.9 and 15.0% in 2018/2019 and 2019/2020, respectively, compared to the control. Leafy spraying with nP-2 further increased these growth and yield traits and iWUE on average by 7.4 and 9.3%, 16.3 and 21.3%, 20.9 and 15.0%, 22.2 and 21.8%, 18.6 and 15.8%, 7.9 and 6.7%, and 30.3 and 31.6% in both seasons, respectively, compared to the control. 

The combination treatments (dI regimes + nP types) displayed significant positive influences on fenugreek plant growth and yield traits (Table 3 and Table 4). The best findings were obtained by the combination treatment of dI-00 (full irrigation regime) + nP-2, which increased PHt, RLh, PDWt, PNpP, SWpP, SYpH, and iWUE on average by 6.0 and 7.2%, 14.5 and 18.2%, 15.2 and 12.6%, 20.0 and 19.2%, 20.1 and 17.1%, 7.9 and 4.9%, and 39.0 and 41% in 2018/2019 and 2019/2020, respectively, compared to the normal control (dI-00 + no foliar spraying with nP). The combination treatment of dI-20 + nP-2 collected growth and yield values closest to those obtained with the normal control, however, it conferred the highest iWUE values with increases of 50.8 and 54.7% in both seasons, respectively, compared to the normal control.

### 3.2. Effect of Irrigation Deficiency and Nanophosphorus on Cell Integrity and Photosynthetic Pigment Contents

The dI-20 and dI-40 and/or leafy nourishment with nP affected fenugreek cell integrity and leaf contents of photosynthetic pigments, osmoprotectants, and P, as well as seed content of P (Table 5 and Table 6).

The dI-20 significantly decreased relative water content (RWC), membrane stability index (MSI), total chlorophyll content (TChC), total carotenoids content (TCrC), leaf phosphorus content (LPC), and seed phosphorus content (SPC) on average by 4.9 and 4.3%, 8.0 and 8.9%, 12.9 and 13.9%, 11.6 and 10.5%, 10.5 and 7.0%, 6.5 and 4.9%, and 2.9 and 3.4% in 2018/2019 and 2019/2020, respectively, while total free amino acids content (TFAa), total soluble sugars content (TSs), and free proline content (FPrC) were increased on average by 52.0 and 56.0%, 71.0 and 74.0%, and 41.2 and 33.3% respectively, when compared to the control. The dI-40 further decreased RWC, MSI, TChC, TCrC, LPC, and SPC on average by 9.8 and 10.1%, 15.7 and 16.8%, 23.9 and 25.5%, 23.3 and 24.4%, 15.8 and 21.1%, and 8.3 and 8.6% in 2018/2019 and 2019/2020, respectively, while TFAa, TSs, and FPrC were increased on average by 180.0 and 196.0%, 146.2 and 143.8%, 17.7 and 16.4%, and 82.4 and 83.3%, respectively, compared to the control.

Under all irrigation regimes, leaf nourishment with nP-1 significantly increased RWC, MSI, TChC, TCrC, LPC, SPC, TFAa, TSs, and FPrC on average by 2.3 and 1.5%, 2.2 and 2.8%, 2.2 and 3.6%, 4.1 and 4.1%, 4.0 and 6.1%, 11.5 and 13.7%, 2.8 and 3.3%, 22.9 and 25.0%, 10.9 and 14.2%, and 14.3 and 8.7% in 2018/2019 and 2019/2020, respectively, when compared to the control. However, all these tested parameters were further increased on average by 4.2 and 3.3%, 4.7 and 5.3%, 5.8 and 8.0%, 8.2 and 9.6%, 6.0 and 10.2%, 17.3 and 19.6%, 5.3 and 5.6%, 57.1 and 58.3%, 37.7 and 38.3%, and 28.6 and 17.4% in both seasons, respectively, with applying nP-2 compared to the control.

The combination treatments (dI regimes + nP types) revealed significant positive influences on fenugreek plant cell integrity and leaf contents of photosynthetic pigments, osmoprotectants, and P, as well as seed content of P (Table 5 and Table 6). For cell integrity and leaf contents of photosynthetic pigments and P, as well as seed content of P, the best findings were obtained by the combination treatment of dI-00 + nP-2, which increased RWC, MSI, TChC, TCrC, LPC, and SPC on average by 5.4 and 2.3%, 4.4 and 5.3%, 5.1 and 6.9%, 6.0 and 4.8%, 7.3 and 9.3%, 14.0 and 21.8%, and 4.0 and 5.3% in 2018/2019 and 2019/2020, respectively, compared to the normal control. However, the combined treatment of dI-20 + nP-2 conferred values closest to those obtained with the normal control. For TFAa, TSs, and FPrC, although the combined treatment of dI-20 + nP-2 conferred greater values than those obtained with the normal control, the combination treatment of dI-40 + nP-2 conferred the greatest findings compared to all combination treatments, including the control.

### 3.3. Effect of Irrigation Deficiency and Nanophosphorus on Antioxidant Activity and Secondary Metabolite Contents

The dI-20 and dI-40 and/or leafy nourishment with nP affected fenugreek plant antioxidant activities and secondary metabolite contents (Table 7 and Table 8).

The dI-20 significantly increased leaf contents of ascorbate (AsA), glutathione (GSH), antioxidant activity (AAc), total phenolics (TPhs), and total flavonoids (TFvs) on average by 26.8 and 24.9%, 15.6 and 23.1%, 23.7 and 22.7%, 9.8 and 14.5%, and 9.7 and 10.5% and increased further with the dI-40 on average by 40.8 and 31.5%, 26.7 and 32.7%, 34.9 and 35.3%, 29.0 and 32.3%, and 16.0 and 15.5% in 2018/2019 and 2019/2020, respectively, compared to the control.

Under all irrigation regimes, leaf nourishment with nP-1 significantly increased leaf contents of AsA, GSH, AAc, TPhs, and TFvs on average by 20.1 and 25.9%, 13.0 and 14.8%, 12.9 and 11.6%, 9.1 and 11.5%, and 8.4 and 7.7% and increased further with applying nP-2 on average by 44.8 and 44.8%, 21.7 and 27.8%, 25.0 and 23.0%, 18.2 and 22.9%, and 17.1 and 15.8% in both seasons, respectively, compared to the control.

The combination treatments (dI regimes + nP types) revealed significant positive influences on fenugreek plant antioxidant activities and secondary metabolite contents (Table 7 and Table 8). Although the combined treatment of dI-20 + nP-2 conferred greater values than those obtained with the normal control, the combination treatment of dI-40 + nP-2 conferred the greatest AsA, GSH, AAc, TPhs, and TFvs findings compared to all combination treatments, including the control.

### 3.4. Effect of Irrigation Deficiency and Nanophosphorus on Leafy Content of the Medicinal Compound Trigonelline

The dI-20 and dI-40 and/or leafy nourishment with nP affected fenugreek leafy content of the medicinal compound trigonelline (Table 8).

The dI-20 significantly increased the leaf trigonelline content on average by 2.4 and 15.6% compared to the dI-40, which in turn increased the trigonelline content on average by 7.8 and 0.4% in 2018/2019 and 2019/2020, respectively, compared to the control. Therefore, the dI-20 significantly increased the leaf trigonelline content on average by 10.4 and 16.0% compared to the control. 

Under all irrigation regimes, leaf nourishment with nP-1 significantly increased the leaf trigonelline content on average by 11.6 and 11.3%, and further increased with nP-2 on average by 19.8 and 19.7% in both seasons, respectively, compared to the control.

The combination treatments (dI regimes + nP types) indicated significant positive influences on the trigonelline content of fenugreek leaves (Table 8). The combined treatment of dI-20 + nP-2 conferred the greatest leaf trigonelline content compared to all combination treatments, including the control (on average by 28.1 and 36.3%).

### 3.5. Effect of Irrigation Deficiency and NanoPhosphorus on Stem and Leaf Anatomy

**Figure 3:**
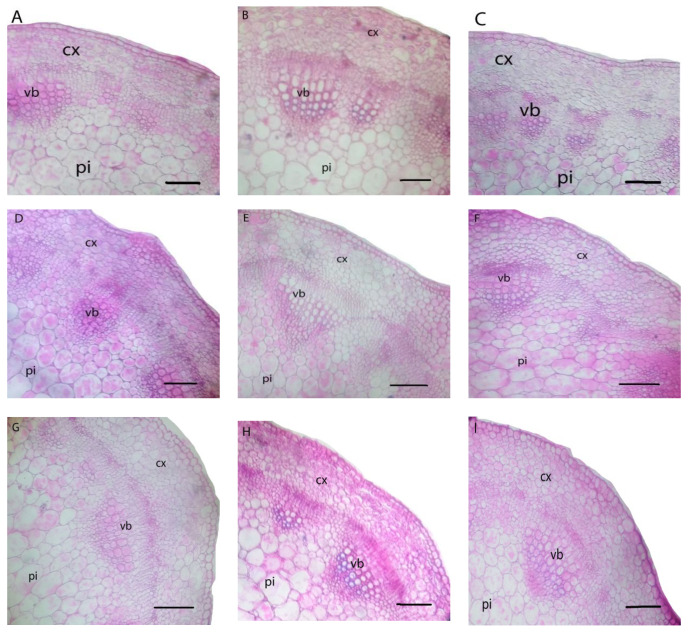
Stem anatomy of fenugreek plants grown under different irrigation levels and phosphorus nanoparticles treatments. (**A**) dI-00 × cP; (**B**) dI-00 × nP-1; (**C**) dI-00 × nP-2; (**D**) dI-20 × cP; (**E**) dI-20 × nP-1; (**F**) dI-20 × nP-2; (**G**) dI-40 × cP; (**H**) dI-40 × nP-1; (**I**) dI-40 × nP-2; (cx = cortex, vb = vascular bundle and pi = pith). Scale bar = 140 µ.

**Figure 4:**
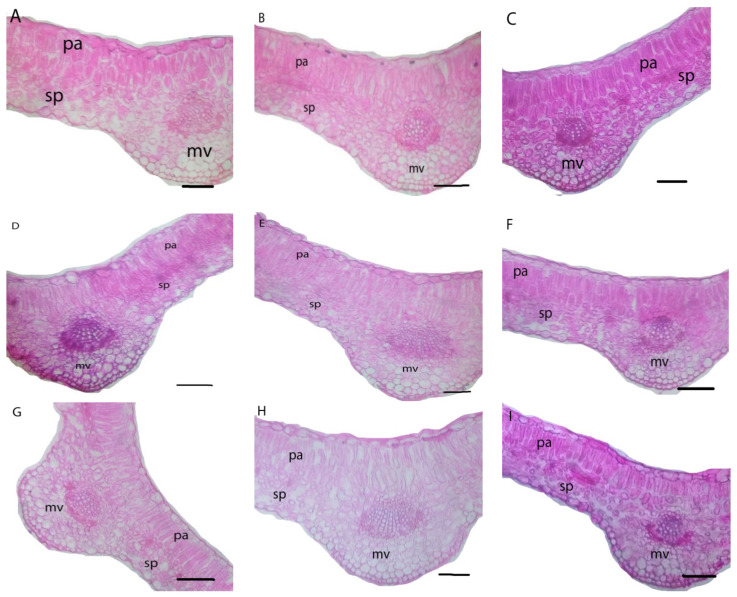
Leaf anatomy of fenugreek plants grown under different irrigation levels and phosphorus nanoparticles treatments. (**A**) dI-00 × cP; (**B**) dI-00 × nP-1; (**C**) dI-00 × nP-2; (**D**) dI-20 × cP; (**E**) dI-20 × nP-1; (**F**) dI-20 × nP-2; (**G**) dI-40 × cP; (**H**) dI-40 × nP-1; (**I**) dI-40 × nP-2; (mv = midvein, pa = palisade tissue and sp = spongy tissue). Scale bar = 140 µ.

The dI-20 and dI-40 and/or leafy nourishment with nP affected fenugreek stem and leaf anatomy (Table 9 and Table 10; Figure 3 and Figure 4).

Although the dI-20 did not affect the stem anatomical features, it significantly reduced the leaf anatomical features; midvein length (MidL), midvein width (MidW), vascular bundle length (VBuL), vascular bundle width (VBuW), number of xylem vessels (NoXyV), lamina thickness (LamTh), palisade tissue thickness (PalTiTh), and spongy tissue thickness (SpTiTh) on average by 7.5, 8.3, 18.9, 11.1, 13.2, 11.2, 15.9, and 26.4%, respectively, compared to the control. The dI-40 significantly reduced both the stem and leaf anatomical features. It decreased stem anatomical features; cortex thickness (CorTh), xylem vessels zone thickness (XyVZTh), number of xylem vessels (NoXyV), diameter of xylem vessels (DXyV), pith length (PiL), and pith width (PiW) on average by 14.9, 11.0, 23.2, 17.4, 43.8, 33.3, 12.4 and 14.3%, and reduced leaf anatomical features; MidL, MidW, VBuL, VBuW, NoXyV, LamTh, PalTiTh, and SpTiTh on average by 21.3, 12.5, 33.1, 29.8, 39.5, 24.5, 36.1, and 33.6%, respectively, compared to the control.

Under all irrigation regimes, leaf nourishment with nP-1 significantly increased the stem anatomical features; CorTh, XyVZTh, NoXyV, PiL, and PiW on average by 6.9, 10.0, 8.2, 6.0, 12.9, 3.4, and 3.9%, and increased the leaf anatomical features; MidL, MidW, VBuL, VBuW, NoXyV, LamTh, PalTiTh, and SpTiTh on average by 10.6, 4.7, 20.0, 9.7, 17.9, 2.5, 5.7, and 20.55%, respectively, compared to the control. Furthermore, leafy nourishment with nP-2 significantly increased the stem anatomical features; CorTh, XyVZTh, NoXyV, PiL, and PiW on average by 22.7, 0.0, 5.7, 9.3, 15.0, 10.7, 16.4, and 14.8%, and increased the leaf anatomical features; MidL, MidW, VBuL, VBuW, NoXyV, LamTh, PalTiTh, and SpTiTh on average by 18.2, 9.4, 26.4, 24.0, 17.9, 10.4, 21.5, and 41.0%, respectively, compared to the control.

The combination treatments (dI regimes + nP types) stated significant positive influences on fenugreek stem and leaf anatomical features (Table 9 and Table 10; Figure 3 and Figure 4). The combination treatment of dI-20 + nP-2 stimulated stem and leaf anatomical features closest to those obtained with the normal control.

## 4. Discussion

Agricultural soils located in dry (semi-arid and arid) regions tend to be alkaline in general, and therefore poor in their structure and nutrients, especially phosphorus (P) due to its fixation [24]. The soil used in this study (Table 1) is poor in P (5.1 mg kg^−1^), which necessitates the use of P, especially at the nanoscale (nP), as a foliar spray to compensate the plant for its inability to obtain P from this soil, especially under conditions of deficit irrigation (dI) stress.

The observations noticed in Table 3, Table 4, Table 5, Table 6, Table 7, Table 8, Table 9 and Table 10 of this study indicate that two levels of dI (a deficit of 20 and 40% of crop evapotranspiration; dI-20 and dI-40) resulted in reductions at varying degrees in fenugreek plant growth and seed yield, leaf integrity measured as relative water content (RWC) and membrane stability index (MSI), photosynthetic pigment contents (PhPiC), leaf and seed contents of P (LSPC), and anatomical features of stem and leaves (LSAnF). However, as noticed in Table 4, Table 5, Table 6, Table 7, Table 8, Table 9 and Table 10, irrigation water use efficiency (iWUE), contents of osmoprotective substances (OPrSC) (e.g., free amino acids; FAaC, soluble sugars; SsC, proline; ProC, and trigonelline; TrigC), antioxidant levels (e.g., ascorbate; AsA and glutathione; GSH), antioxidative activity (AAc), and contents of secondary metabolites (e.g., phenolics compounds; PhCC and flavonoids; FlaC) were increased at varying degrees under both the dI-20 and dI-40. The increases obtained in iWUE, OPrSC, TrigC, AsA, GSH, AAc, PhCC, and FlaC are various mechanisms that plants utilized in this study to ameliorate their growth and seed production at acceptable levels under dI stress. This positive finding required positive modulations in RWC, MSI, PhPiC, LSPC, and LSAnF, along with further improvements in OPrSC, TrigC, AsA, GSH, AAc, PhCC, and FlaC, which were already achieved through foliar nourishment with nP (Table 3, Table 4, Table 5, Table 6, Table 7, Table 8, Table 9 and Table 10). These results are consistent with those obtained previously [10,16,24,37,39,46,47,69,70,71,72,73,74].

As previously reported in countless research papers, P is very important for all aspects of plant growth and development, and yield quality of plant products as it is a major component of ATP, RNA, DNA, and cell membrane phospholipids. Besides, P improves the translocations of photosynthates to edible parts and prolongs the photosynthetic period due to the longest stay-green feature, which contributes to an increase in the seed filling duration under stress. It supports the plant’s ability to maintain leaf integrity and contributes to improving the system of antioxidant defenses under stress. It also promotes cell expansions and meristematic cell activities due to its participation in the increase of OPrSC [24,75,76]. However, some reports showed superior performance of nP over conventional phosphorus (cP) in restoring plant growth and productivity under stress conditions [24,77]. These reports considered nP as an effective source of P nutrient as soluble P fertilizer because plants can absorb sufficient amount of nP when applied as foliar fertilization.

This is the first study, in which foliar nourishment was applied with nP at two nanoscales (25 and 50 nm) versus cP for dI-stressed fenugreek plants. The interesting thing observed in this study was that the smaller nP (25 nm) outperformed the larger nP scale (50 nm), which in turn outperformed the cP in increasing all parameters examined for dI-stressed fenugreek plants (Table 3, Table 4, Table 5, Table 6, Table 7, Table 8, Table 9 and Table 10). This result can be attributed to that smaller nP can penetrate and reach vital function sites faster than larger nP scale.

Despite the increases in iWUE (Table 4), fenugreek plants were not able to grow and produce seeds well under dI-20 stress, and the inability to perform was more pronounced under dI-40 stress conditions (Table 3). These adverse growth results under dI stress could be due to the reduced relative turgidity (RWC), MSI (Table 5), P content (Table 6), and/or dehydration of the protoplasm of leaf tissue cells, resulting in a decrease in the chlorophyll and carotenoid contents (PhPiC), photosynthesis activity (Table 5), cell division, and cell enlargement [16,25,72], and plant anatomical features (Table 9 and Table 10). Besides, attacking the protoplasm, which is the stage of vital processes in the cell, through reactive oxygen species (ROS) generated by dI stress, disrupting the growth processes [25]. However, compared to cP, providing dI-stressed fenugreek plants with nP as foliar nourishment enabled the plants to develop/adopt potential mechanisms to increase their tolerance to dI stress, and thus the plants performed better in favor of their growth. These findings are in good agreement with those in Rady et al. [24] and El-Ghany et al. [78], who demonstrated that foliar nourishment of plants with nP is better than cP. The growth promotion can be attributed to that nP particles easily penetrate from stomata into leaf tissue cells to perform advantageous influences due to their higher reactivity. The greater density of reactive zones and more specific surface area, which provides more sites for different vital metabolic processes in the plant system, leads to an increase in the nP higher reactivity [19,24,26]. The higher surface areas and penetrability make the nP a more efficient product (namely P utilization efficiency) compared to cP resulting in improved growth and seed production of fenugreek plants, especially under dI stress. Besides, compared with nP, the better returns (growth, seed yield, and higher P content) of fenugreek plants supplemented with nP appeared to be due to the improvement in the translocation of photosynthates from leaves to pods as a result of long periods of stay-green feature, photosynthesis, and seed filling. Also, chlorophyll biosynthesis and positioning, and CO_2_ uptake and assimilation appeared to increase due to the increase in leaf integrity (RWC and MSI) as a result of nP application (Table 5). Since cell membranes mainly contain phospholipids, nourishing fenugreek plants with nP can facilitate P access to these membranes to increase their stability. These important mechanisms of nP increased plant growth and endogenous P content, reflecting in the increased seed yield and seed content of P (Table 3, Table 4 and Table 6). The results of improvement in plant seed yield are in agreement with those of author et al. [15,16,25,39,72,73].

Under dI stress, in this study, although the contents of compatible osmolyte compounds (free amino acids, soluble sugars, proline, and trigonelline), antioxidants (AsA, GSH, phenolics, and flavonoids) were increased, they were not sufficient for fenugreek plants to defend themselves against stress to perform acceptable growth and seed yield. However, foliar nourishment with nP endowed the plants with more contents of these osmoprotectants and antioxidants with more antioxidative activity (Table 6, Table 7 and Table 8). These further improvements conferred adaptive mechanisms, including maintaining proper cellular turgor in favor of the plant physiological functions [24,25], which enabled plants to defend themselves well against stress and led to acceptable growth and seed yield (Table 3 and Table 4). The findings of this study indicated a potent relationship between the nP-induced increase in plant root length along with the dry matter (Table 3) due to the increase in osmoprotective substances (Table 6) and the yield of productive seeds (Table 4). For better seed production, the dI-stressed fenugreek plants required balanced osmotic adjustment, which was achieved by increasing the osmoprotectants through the application of nP (Table 4 and Table 6). Also, they required a strong antioxidant system for more antioxidative activity [25], which was also achieved with nP nourishment (Table 7 and Table 8).

Previous reports [24,29] show the importance of increasing antioxidants and antioxidant activity to strengthen the antioxidant defense system within the plant so that it can give reasonable growth and yield. This is what happened, in this study, with the use of nP for fenugreek plants growing under dI stress (Table 6, Table 7 and Table 8). The increase in antioxidants (AsA, GSH, phenolics, and flavonoids) and their antioxidant activity neutralizes ROS by scavenging more of them, along with the elevated osmoprotectants, including trigonelline to enable plants to grow well under dI stress [24,29]. In response to dI stress conditions, in this study, trigonelline accumulated under dI stress and accumulated more with nP application (Table 8). 

As previously reported [10,13], trigonelline accumulates to function as an osmoprotective compound, as it migrates from seed cotyledons to other plant tissues during the establishment of early seedlings to exert its physiological influence. It protects organisms/plants against abiotic stresses via osmoregulation and osmoprotection [10,13,14]. Therefore, trigonelline as an osmoprotective compound is associated in modulating fenugreek plant physiological and biochemical responses, and improving plant metabolic processes, causing an increase in P content in both leaves and seeds under dI × nP application. Our results are supported by the findings of author et al. [10,13,14,15,16,70,72].

With the use of nP foliar nourishment, anatomical features of fenugreek stem and leaves were responsive to dI (Table 9 and Table 10; Figure 2 and Figure 3). This finding could be achieved as plant adaptive mechanisms under limited water supply to regulate the conductivity of water flow pathways along with the soil-plant-atmosphere system. Previous anatomical results [19,79,80] are in agreement with our results. In this study, the anatomical modulation under stress encouraged a stable release of P nutrient at its reactive sites and a steady uptake of water (RWC) and nutrients (including P). This facilitates plant growth under stress due to increased plant’s metabolic efficiency, which was reflected in satisfactory seed yield.

## 5. Conclusions

Foliar nourishment with nanophosphorus can be used as an innovative technology strategy to enhance growth and seed yield, as well as the physiological, biochemical, and anatomical responses of fenugreek plants grown under deficit irrigation stress conditions, especially in soils located in dry environments. Such soils suffer from a lack of nutrients, including phosphorus. Under deficit irrigation stress, to obtain an adequate seed yield, plants must maintain a higher cellular water content along with a higher content of antioxidants (increased antioxidative activity) and osmoprotectants, including trigonelline compound, all were achieved by foliar feeding with nanophosphorus. Additionally, the interesting thing about this study was that the smaller (25 nm) nanophosphorus performed well compared to the larger (50 nm) nanophosphorus, which in turn outperformed the conventional soil-applied phosphorus. Therefore, a viable recommendation for using nanophosphorus for soils lacking phosphorous in dry areas, in agriculture. This nanostrategy controls nutrient release and can be an effective agro-management for sustainable agriculture, including fenugreek production under deficient irrigation conditions, and environmental conservation. Nanofertilizers potentially help reduce the amounts of fertilizers added to crops through foliar spraying, minimize nutrient losses and production costs, and maximize nutrient-use efficiency.

## Figures and Tables

**Table 1:** The main physical and chemical properties of the soil used for experiments.

Depth (cm)	Particle Size Distribution				*ρ_b_* (g cm^−3^)	K_sat_ cm h^−1^	θ_Fc_ (%)	θ_WP_ (%)	AW (%)
	Sand %	Silt %	Clay %	Texture Class					
0–25	9.9	20.0	70.1	Clay	1.41	1.15	35.1	19.6	15.5
25–50	8.6	20.4	71.0	Clay	1.37	1.02	33.3	19.1	14.1
Mean	9.2	20.2	70.6	Clay	1.39	1.09	34.2	19.4	14.8
Chemical properties					Value				
pH [at a soil: water (*w*/*v*) ratio of 1:2.5]					7.55				
ECe (dS.m^−1^; soil paste extract)					2.57				
CEC (cmole kg^−1^)					14.2				
CaCO_3_ (g kg^−1^)					4.81				
OM (%)					1.20				
Available		N	mg kg^−1^ soil		52.6				
		P			5.10				
		K			59.9				

*ρ_b_* = Bulk density, K_sat_ = Hydraulic conductivity, θ_Fc_ = Volumetric water content at field capacity, θ_WP_ = Volumetric water content at wilting point, and AW = Available water.

**Table 2:** IWA for each imposed stress in both experimental seasons.

Irrigation Treatments	2018/2019		2019/2020	
	per ha (10,000 m^2^)	per Plot (6 m^2^)	per ha (10,000 m^2^)	per Plot (6 m^2^)
Control (dI-00)	4500 m^3^	2.70 m^3^	4460 m^3^	2.68 m^3^
dI-20	3600 m^3^	2.16 m^3^	3568 m^3^	2.14 m^3^
dI-40	2700 m^3^	1.62 m^3^	2676 m^3^	1.61 m^3^

**Table 3:** Growth traits of fenugreek plants as affected by different irrigation water regimes and soil and foliar nourishing with phosphorus (P) during two growing seasons.

Treatments		PHt (cm)		RLh (cm)		PDWt (g)	
		S-I	S-II	S-I	S-II	S-I	S-II
dI-00	cP	70.0 ^c^ ± 1.4	70.1 ^c^ ± 1.4	20.5 ^c^ ± 0.7	19.3 ^d^ ± 0.7	16.9 ^c^ ± 0.5	17.1 ^c^ ± 0.5
	nP-1	72.7 ^b^ ± 1.4	73.4 ^b^ ± 1.5	22.2 ^b^ ± 0.7	21.9 ^b^ ± 0.7	18.5 ^b^ ± 0.5	18.3 ^b^ ± 0.5
	nP-2	74.2 ^a^ ± 1.5	75.2 ^a^ ± 1.5	23.5 ^a^ ± 0.7	22.8 ^a^ ± 0.7	19.5 ^a^ ± 0.5	19.3 ^a^ ± 0.5
dI-20	cP	63.2 ^f^ ± 1.2	62.2 ^f^ ± 1.1	16.4 ^f^ ± 0.6	16.8 ^f^ ± 0.6	14.1 ^f^ ± 0.4	13.9 ^f^ ± 0.4
	nP-1	65.5 ^e^ ± 1.3	64.7 ^e^ ± 1.3	17.4 ^e^ ± 0.6	18.8 ^e^ ± 0.6	15.7 ^e^ ± 0.5	15.1 ^e^ ± 0.4
	nP-2	67.0 ^d^ ± 1.3	67.7 ^d^ ± 1.3	19.5 ^d^ ± 0.6	20.8 ^c^ ± 0.7	16.8 ^cd^ ± 0.5	16.0 ^d^ ± 0.5
dI-40	cP	54.0 ^i^ ± 1.1	53.4 ^i^ ± 1.0	13.8 ^h^ ± 0.5	12.5 ^i^ ± 0.5	10.5 ^i^ ± 0.4	10.9 ^i^ ± 0.4
	nP-1	57.9 ^h^ ± 1.3	56.2 ^h^ ± 1.0	14.7 ^gh^ ± 0.5	13.6 ^h^ ± 0.5	11.6 ^h^ ± 0.4	12.1 ^h^ ± 0.4
	nP-2	59.8 ^g^ ± 1.3	60.2 ^g^ ± 1.3	15.9 ^g^ ± 0.6	15.2 ^g^ ± 0.6	13.9 ^fg^ ± 0.4	13.0 ^g^ ± 0.4
*p*-value		1.1	1.3	1.0	0.4	0.2	0.5

Means ± SE followed by the same letter in each column are not significantly different according to the LSD test (*p* ≤ 0.05). PHt is the plant height, RLh is the root length, PDWt is the dry weight plant^−1^, S-I and S-II are the first (2018/2019) and second (2019/2020) seasons, respectively, dI-00 is the irrigation by 100% of crop evapotranspiration (ETc), dI-20 is the irrigation by 80% of ETc, and dI-40 is the irrigation by 60% of ETc, cP is the conventional soil addition of phosphorus (400 kg P ha^−1^; 15.5% P_2_O_5_), nP-1 is nanophosphorus with 10.8–14.7 nm that was foliarly applied at 0.1 g L^−1^, nP-2 is nanophosphorus with 4.9–8.6 nm that was foliarly applied at 0.1 g L^−1^.

**Table 4:** Yield and yield components, and water use efficiency of fenugreek plants as affected by different irrigation water regimes and soil and foliar nourishing with phosphorus (P) during two growing seasons.

Treatments		PNpP		SWpP (g)		SY (t ha^−^^1^)		iWUE (kg Seed Yield m^−^^3^ Water)	
		S-I	S-II	S-I	S-II	S-I	S-II	S-I	S-II
dI-00	cP	12.7 ^c^ ± 0.3	12.4 ^c^ ± 0.4	4.33 ^c^ ± 0.26	4.50 ^c^ ± 0.26	6.07 ^c^ ± 0.36	6.12 ^c^ ± 0.35	1.18 ^e^ ± 0.02	1.17 ^d^ ± 0.03
	nP-1	13.8 ^b^ ± 0.4	13.6 ^b^ ± 0.4	4.67 ^b^ ± 0.26	4.82 ^b^ ± 0.27	6.26 ^b^ ± 0.37	6.28 ^b^ ± 0.37	1.39 ^d^ ± 0.03	1.41 ^c^ ± 0.03
	nP-2	15.2 ^a^ ± 0.4	14.8 ^a^ ± 0.4	5.20 ^a^ ± 0.28	5.27 ^a^ ± 0.29	6.55 ^a^ ± 0.38	6.42 ^a^ ± 0.37	1.64 ^b^ ± 0.04	1.65 ^b^ ± 0.04
dI-20	cP	9.7 ^f^ ± 0.3	10.0 ^f^ ± 0.4	3.22 ^f^ ± 0.23	3.66 ^f^ ± 0.24	5.50 ^f^ ± 0.33	5.54 ^f^ ± 0.34	1.40 ^d^ ± 0.03	1.41 ^c^ ± 0.03
	nP-1	10.4 ^e^ ± 0.4	11.1 ^e^ ± 0.4	3.44 ^e^ ± 0.24	4.07 ^e^ ± 0.25	5.70 ^e^ ± 0.35	5.74 ^e^ ± 0.34	1.56 ^bc^ ± 0.04	1.59 ^b^ ± 0.05
	nP-2	11.9 ^d^ ± 0.4	11.7 ^d^ ± 0.4	3.91 ^d^ ± 0.25	4.15 ^d^ ± 0.26	5.91 ^d^ ± 0.35	5.96 ^d^ ± 0.35	1.78 ^a^ ± 0.04	1.81 ^a^ ± 0.06
dI-40	cP	6.6 ^i^ ± 0.3	6.0 ^i^ ± 0.3	2.60 ^i^ ± 0.23	2.47 ^i^ ± 0.22	4.02 ^i^ ± 0.30	4.10 ^i^ ± 0.33	1.39 ^d^ ± 0.03	1.42 ^c^ ± 0.03
	nP-1	7.8 ^h^ ± 0.3	6.9 ^h^ ± 0.3	2.88 ^gh^ ± 0.24	2.85 ^h^ ± 0.24	4.25 ^h^ ± 0.32	4.33 ^h^ ± 0.34	1.53 ^c^ ± 0.03	1.58 ^b^ ± 0.03
	nP-2	8.7 ^g^ ± 0.3	8.2 ^g^ ± 0.3	2.92 ^g^ ± 0.24	2.91 ^g^ ± 0.24	4.38 ^g^ ± 0.33	4.41 ^g^ ± 0.35	1.75 ^a^ ± 0.06	1.80 ^a^ ± 0.05
*p*-value		0.40	0.50	0.04	0.05	0.10	0.07	0.04	0.08

Means ± SE followed by the same letter in each column are not significantly different according to the LSD test (*p* ≤ 0.05). NpP is the pods No. plant^−1^, SWpP is the seed weight plant^−1^, SY is the seed yield, iWUE is the irrigation water use efficiency, S-I and S-II are the first (2018/2019) and second (2019/2020) seasons, respectively, dI-00 is the irrigation by 100% of crop evapotranspiration (ETc), dI-20 is the irrigation by 80% of ETc, and dI-40 is the irrigation by 60% of ETc, cP is the conventional soil addition of phosphorus (400 kg P ha^−1^; 15.5% P_2_O_5_), nP-1 is nanophosphorus with 10.8–14.7 nm that was foliarly applied at 0.1 g L^−1^, nP-2 is nanophosphorus with 4.9–8.6 nm that was foliarly applied at 0.1 g L^−1^.

**Table 5:** Cell integrity and photosynthetic pigment contents of fenugreek plants as affected by different irrigation water regimes and soil and foliar nourishing with phosphorus (P) during two growing seasons.

Treatments		RWC (%)		MSI (%)		TChC (mg g^−^^1^ FW)		TCrC (mg g^−^^1^ FW)	
		S-I	S-II	S-I	S-II	S-I	S-II	S-I	S-II
dI-00	cP	83.1 ^c^ ± 1.2	84.3 ^c^ ± 1.1	70.1 ^c^ ± 0.7	69.6 ^c^ ± 0.8	2.41 ^b^ ± 0.13	2.43 ^c^ ± 0.13	0.55 ^b^ ± 0.04	0.54 ^c^ ± 0.03
	nP-1	85.6 ^b^ ± 1.3	85.0 ^b^ ± 1.1	71.4 ^b^ ± 0.8	72.0 ^b^ ± 0.8	2.51 ^a^ ± 0.13	2.51 ^b^ ± 0.12	0.57 ^ab^ ± 0.4	0.56 ^b^ ± 0.03
	nP-2	87.6 ^a^ ± 1.3	86.3 ^a^ ± 1.3	73.2 ^a^ ± 0.9	73.3 ^a^ ± 0.9	2.54 ^a^ ± 0.14	2.58 ^a^ ± 0.13	0.59 ^a^ ± 0.04	0.59 ^a^ ± 0.03
dI-20	cP	80.2 ^f^ ± 1.0	80.6 ^f^ ± 0.9	64.5 ^f^ ± 0.8	63.8 ^f^ ± 0.7	2.13 ^d^ ± 0.12	2.09 ^f^ ± 0.13	0.49 ^d^ ± 0.03	0.50 ^d^ ± 0.02
	nP-1	81.7 ^e^ ± 1.1	81.6 ^e^ ± 0.9	65.9 ^e^ ± 0.8	65.3 ^e^ ± 0.7	2.17 ^d^ ± 0.13	2.19 ^e^ ± 012	0.52 ^c^ ± 0.03	0.54 ^c^ ± 0.03
	nP-2	82.0 ^d^ ± 1.1	82.4 ^d^ ± 1.0	67.1 ^d^ ± 0.8	66.7 ^d^ ± 0.7	2.26 ^c^ ± 0.13	2.31 ^d^ ± 0.13	0.53 ^bc^ ± 0.03	0.55 ^bc^ ± 0.03
dI-40	cP	75.3 ^i^ ± 0.8	74.6 ^i^ ± 0.8	58.7 ^i^ ± 0.7	58.0 ^i^ ± 0.6	1.82 ^g^ ± 0.10	1.82 ^i^ ± 0.10	0.45 ^ef^ ± 0.02	0.44 ^f^ ± 0.02
	nP-1	76.7 ^h^ ± 0.9	76.6 ^h^ ± 0.9	60.3 ^h^ ± 0.7	59.4 ^h^ ± 0.7	1.88 ^f^ ± 0.11	1.88 ^h^ ± 0.11	0.46 ^e^ ± 0.02	0.46 ^e^ ± 0.02
	nP-2	79.1 ^g^ ± 1.0	78.7 ^g^ ± 0.9	62.1 ^g^ ± 0.8	61.6 ^g^ ± 0.7	1.98 ^e^ ± 0.11	1.96 ^g^ ± 0.11	0.48 ^de^ ± 0.03	0.47 ^e^ ± 0.02
*p*-value		0.3	0.6	0.9	1.1	0.05	0.06	0.03	0.02

Means ± SE followed by the same letter in each column are not significantly different according to the LSD test (*p* ≤ 0.05). RWC is the relative water content, MSI is the membrane stability index, TChC is the total chlorophyll content, TCrC is the total carotenoids content, S-I and S-II are the first (2018/2019) and second (2019/2020) seasons, respectively, dI-00 is the irrigation by 100% of crop evapotranspiration (ETc), dI-20 is the irrigation by 80% of ETc, and dI-40 is the irrigation by 60% of ETc, cP is the conventional soil addition of phosphorus (400 kg P ha^−1^; 15.5% P_2_O_5_), nP-1 is nanophosphorus with 10.8–14.7 nm that was foliarly applied at 0.1 g L^−1^, nP-2 is nanophosphorus with 4.9–8.6 nm that was foliarly applied at 0.1 g L^−1^.

**Table 6:** Total free amino acids (TFAa), total soluble sugars (TSs), and free proline contents of fenugreek plants as affected by different irrigation water regimes and soil and foliar nourishing with phosphorus (P) during two growing seasons.

Treatments		TFAa Conrent (mg g^−^^1^ DW)		TSs Content (mg g^−^^1^ DW)		Proline Content (μg g^−^^1^ DW)		Leaf P (mg g^−^^1^ DW)		Seed P (mg g^−^^1^ DW)	
		S-I	S-II	S-I	S-II	S-I	S-II	S-I	S-II	S-I	S-II
dI-00	cP	0.21 ^i^ ± 0.01	0.22 ^hi^ ± 0.01	0.78 ^i^ ± 0.06	0.85 ^gh^ ± 0.07	0.14 ^g^ ± 0.01	0.16 ^fg^ ± 0.01	0.57 ^d^ ± 0.03	0.55 ^c^ ± 0.03	3.78 ^bc^ ± 0.18	3.75 ^bc^ ± 0.17
	nP-1	0.26 ^h^ ± 0.01	0.24 ^h^ ± 0.01	0.87 ^h^ ± 0.06	0.90 ^g^ ± 0.07	0.17 ^ef^ ± 0.01	0.17 ^f^ ± 0.01	0.63 ^ab^ ± 0.04	0.62 ^b^ ± 004	3.82 ^b^ ± 0.18	3.86 ^ab^ ± 0.19
	nP-2	0.29 ^g^ ± 0.02	0.28 ^g^ ± 0.01	1.13 ^g^ ± 0.08	1.14 ^f^ ± 0.07	0.19 ^e^ ± 0.01	0.20 ^e^ ± 0.01	0.65 ^a^ ± 0.04	0.67 ^a^ ± 0.04	3.93 ^a^ ± 0.20	3.95 ^a^ ± 0.21
dI-20	cP	0.33 ^f^ ± 0.02	0.34 ^f^ ± 0.02	1.22 ^f^ ± 0.08	1.26 ^e^ ± 0.08	0.22 ^d^ ± 0.01	0.22 ^e^ ± 0.01	0.53 ^ef^ ± 0.02	0.52 ^c^ ± 0.03	3.62 ^d^ ± 0.17	3.59 ^d^ ± 0.17
	nP-1	0.37 ^e^ ± 0.02	0.38 ^e^ ± 0.02	1.52 ^e^ ± 0.08	1.65 ^d^ ± 0.08	0.24 ^d^ ± 0.01	0.25 ^d^ ± 0.01	0.59 ^cd^ ± 0.03	0.60 ^b^ ± 0.03	3.72 ^c^ ± 0.18	3.74 ^c^ ± 0.19
	nP-2	0.44 ^d^ ± 0.03	0.45 ^d^ ± 0.02	2.04 ^d^ ± 0.08	2.10 ^c^ ± 0.08	0.27 ^c^ ± 0.01	0.26 ^d^ ± 0.01	0.61 ^b^^c^ ± 0.04	0.62 ^b^ ± 0.04	3.84 ^ab^ ± 0.20	3.83 ^bc^ ± 0.19
dI-40	cP	0.50 ^c^ ± 0.03	0.52 ^c^ ± 0.03	2.15 ^bc^ ± 0.08	2.12 ^c^ ± 0.08	0.28 ^c^ ± 0.01	0.30 ^c^ ± 0.02	0.45 ^g^ ± 0.02	0.47 ^d^ ± 0.02	3.40 ^e^ ± 0.15	3.42 ^e^ ± 0.15
	nP-1	0.67 ^b^ ± 0.03	0.72 ^b^ ± 0.03	2.19 ^b^ ± 0.09	2.29 ^b^ ± 0.08	0.31 ^b^ ± 0.02	0.33 ^b^ ± 0.02	0.51 ^f^ ± 0.03	0.52 ^c^ ± 0.03	3.55 ^d^ ± 0.17	3.54 ^d^ ± 0.16
	nP-2	0.92 ^a^ ± 0.04	0.99 ^a^ ± 0.04	2.52 ^a^ ± 0.08	2.62 ^a^ ± 0.09	0.34 ^a^ ± 0.02	0.36 ^a^ ± 0.02	0.56 ^de^ ± 0.03	0.55 ^c^ ± 0.03	3.61 ^d^ ± 0.17	3.59 ^d^ ± 0.17
*p*-value		0.02	0.03	0.05	0.06	0.03	0.03	0.04	0.04	0.10	0.12

Means ± SE followed by the same letter in each column are not significantly different according to the LSD test (*p* ≤ 0.05). S-I and S-II are the first (2018/2019) and second (2019/2020) seasons, respectively, dI-00 is the irrigation by 100% of crop evapotranspiration (ETc), dI-20 is the irrigation by 80% of ETc, and dI-40 is the irrigation by 60% of ETc, cP is the conventional soil addition of phosphorus (400 kg P ha^−1^; 15.5% P_2_O_5_), nP-1 is nanophosphorus with 10.8–14.7 nm that was foliarly applied at 0.1 g L^−1^, nP-2 is nanophosphorus with 4.9–8.6 nm that was foliarly applied at 0.1 g L^−1^.

**Table 7:** The contents of ascorbate (AsA), glutathione (GSH), and antioxidative activity (AAc) in leaves of fenugreek plants as affected by different irrigation water regimes and soil and foliar nourishing with phosphorus (P) during two growing seasons.

Treatments		AsA (μmole g^−1^ Fresh Leaf)		GSH (μmole g^−1^ Fresh Leaf)		AAc (TE g^−1^ Seed DW)	
		S-I	S-II	S-I	S-II	S-I	S-II
dI-00	cP	1.31 ^g^ ± 0.03	1.42 ^g^ ± 0.04	0.40 ^f^ ± 0.00	0.46 ^g^ ± 0.01	35.8 ^f^ ± 0.7	37.6 ^f^ ± 0.9
	nP-1	1.58 ^f^ ± 0.04	1.81 ^f^ ± 0.05	0.46 ^e^ ± 0.00	0.53 ^f^ ± 0.01	39.7 ^e^ ± 0.9	41.9 ^e^ ± 1.0
	nP-2	1.83 ^e^ ± 0.04	2.20 ^d^ ± 0.06	0.49 ^d^ ± 0.01	0.58 ^de^ ± 0.01	43.2 ^d^ ± 1.2	46.2 ^d^ ± 1.3
dI-20	cP	1.62 ^f^ ± 0.04	1.81 ^f^ ± 0.04	0.47 ^de^ ± 0.01	0.55 ^ef^ ± 0.02	42.8 ^de^ ± 1.1	45.7 ^d^ ± 1.2
	nP-1	1.94 ^d^ ± 0.05	2.32 ^c^ ± 0.06	0.52 ^c^ ± 0.01	0.64 ^c^ ± 0.02	49.1 ^c^ ± 1.5	51.5 ^c^ ± 1.5
	nP-2	2.41 ^b^ ± 0.05	2.64 ^a^ ± 0.07	0.57 ^b^ ± 0.02	0.72 ^b^ ± 0.03	55.2 ^b^ ± 1.8	57.0 ^b^ ± 1.7
dI-40	cP	1.84 ^e^ ± 0.04	1.98 ^e^ ± 0.05	0.52 ^c^ ± 0.01	0.61 ^cd^ ± 0.02	47.5 ^c^ ± 1.6	51.2 ^c^ ± 1.5
	nP-1	2.22 ^c^ ± 0.05	2.44 ^b^ ± 0.06	0.57 ^b^ ± 0.01	0.69 ^b^ ± 0.02	53.4 ^b^ ± 1.8	56.7 ^b^ ± 1.5
	nP-2	2.56 ^a^ ± 0.06	2.71 ^a^ ± 0.07	0.62 ^a^ ± 0.02	0.76 ^a^ ± 0.03	59.2 ^a^ ± 1.9	62.1 ^a^ ± 1.8
*p*-value		0.06	0.09	0.03	0.04	3.10	3.50

Means ± SE followed by the same letter in each column are not significantly different according to the LSD test (*p* ≤ 0.05). S-I and S-II are the first (2018/2019) and second (2019/2020) seasons, respectively, dI-00 is the irrigation by 100% of crop evapotranspiration (ETc), dI-20 is the irrigation by 80% of ETc, and dI-40 is the irrigation by 60% of ETc, cP is the conventional soil addition of phosphorus (400 kg P ha^−1^; 15.5% P_2_O_5_), nP-1 is nanophosphorus with 10.8–14.7 nm that was foliarly applied at 0.1 g L^−1^, nP-2 is nanophosphorus with 4.9–8.6 nm that was foliarly applied at 0.1 g L^−1^.

**Table 8:** The contents of trigonelline, total phenolics (TPhs), and total flavonoids (TFvs) of the seeds produced from fenugreek plants as affected by different irrigation water regimes and soil and foliar nourishing with phosphorus (P) during two growing seasons.

Treatments		Trigonelline Content (μg g^−1^ Dry Seed)		TPhs Content (mg CA g^−1^ Dry Seed)		TFvs Content (mg RU g^−1^ Dry Seed)	
		S-I	S-II	S-I	S-II	S-I	S-II
dI-00	cP	46.3 ^f^ ± 1.2	51.2 ^f^ ± 1.1	2.44 ^f^ ± 0.08	2.39 ^g^ ± 0.06	3.51 ^f^ ± 0.15	4.02 ^e^ ± 0.18
	nP-1	49.9 ^de^ ± 1.4	56.4 ^e^ ± 1.5	2.68 ^e^ ± 0.10	2.70 ^f^ ± 0.11	3.80 ^ef^ ± 0.18	4.42 ^d^ ± 0.22
	nP-2	54.2 ^bc^ ± 1.7	61.3 ^c^ ± 1.8	2.86 ^d^ ± 0.12	2.98 ^e^ ± 0.13	4.12 ^cd^ ± 0.21	4.75 ^cd^ ± 0.30
dI-20	cP	51.2 ^cd^ ± 1.4	60.2 ^cd^ ± 1.7	2.68 ^e^ ± 0.09	2.71 ^f^ ± 0.11	3.84 ^de^ ± 0.17	4.49 ^d^ ± 0.22
	nP-1	55.4 ^b^ ± 1.6	66.0 ^b^ ± 2.0	2.89 ^d^ ± 0.12	3.07 ^e^ ± 0.14	4.22 ^bc^ ± 0.22	4.86 ^bc^ ± 0.30
	nP-2	59.3 ^a^ ± 1.8	69.8 ^a^ ± 2.2	3.18 ^c^ ± 0.15	3.46 ^cd^ ± 0.17	4.48 ^b^ ± 0.25	5.22 ^ab^ ± 0.32
dI-40	cP	46.8 ^ef^ ± 1.1	50.1 ^f^ ± 1.2	3.14 ^c^ ± 0.13	3.28 ^d^ ± 0.15	4.09 ^cde^ ± 0.21	4.78 ^cd^ ± 0.30
	nP-1	55.9 ^b^ ± 1.6	57.4 ^de^ ± 1.2	3.42 ^b^ ± 0.16	3.55 ^bc^ ± 0.17	4.38 ^bc^ ± 0.22	5.04 ^bc^ ± 0.31
	nP-2	59.2 ^a^ ± 1.9	62.0 ^c^ ± 1.7	3.72 ^a^ ± 0.18	3.84 ^a^ ± 0.20	4.79 ^a^ ± 0.28	5.41 ^a^ ± 0.35
*p*-value		3.30	3.50	0.15	0.21	0.30	0.37

Means ± SE followed by the same letter in each column are not significantly different according to the LSD test (*p* ≤ 0.05). S-I and S-II are the first (2018/2019) and second (2019/2020) seasons, respectively, dI-00 is the irrigation by 100% of crop evapotranspiration (ETc), dI-20 is the irrigation by 80% of ETc, and dI-40 is the irrigation by 60% of ETc, cP is the conventional soil addition of phosphorus (400 kg P ha^−1^; 15.5% P_2_O_5_), nP-1 is nanophosphorus with 10.8–14.7 nm that was foliarly applied at 0.1 g L^−1^, nP-2 is nanophosphorus with 4.9–8.6 nm that was foliarly applied at 0.1 g L^−1^.

**Table 9:** Stem anatomical features of fenugreek plants as affected by different irrigation water regimes and soil and foliar nourishing with phosphorus (P) during the season 2019/2020.

Treatments		Length (µ)	Width (µ)	CorTh (µ)	XyVZTh (µ)	NoXyV	DXyV (µ)	PiL (µ)	PiW (µ)
dI-00	cP	2300 ^c^	2575 ^d^	225 ^c^	325 ^b^	760 ^c^	37.5 ^a^	1575 ^c^	1850 ^d^
	nP-1	2625 ^b^	2650 ^b^	250 ^b^	325 ^b^	880 ^b^	37.5 ^a^	1600 ^b^	1925 ^b^
	nP-2	2650 ^a^	2725 ^a^	275 ^a^	350 ^a^	960 ^a^	37.5 ^a^	1650 ^a^	2000 ^a^
dI-20	cP	2200 ^g^	2325 ^g^	225 ^c^	275 ^d^	510 ^fg^	25.0 ^b^	1475 ^f^	1700 ^g^
	nP-1	2250 ^f^	2575 ^d^	225 ^c^	300 ^c^	570 ^e^	25.0 ^b^	1525 ^e^	1750 ^e^
	nP-2	2450 ^d^	2600 ^c^	250 ^b^	325 ^b^	630 ^d^	25.0 ^b^	1550 ^d^	1875 ^c^
dI-40	cP	2000 ^i^	2125 ^h^	175 ^e^	250 ^e^	450 ^h^	25.0 ^b^	1350 ^i^	1575 ^i^
	nP-1	2075 ^h^	2500 ^e^	200 ^d^	275 ^d^	490 ^g^	25.0 ^b^	1425 ^h^	1650 ^h^
	nP-2	2375 ^e^	2450 ^f^	200 ^d^	300 ^c^	520 ^f^	25.0 ^b^	1450 ^g^	1725 ^f^
*p*-value		22	25	11	14	21	1.5	12	15

Means followed by the same letter in each column are not significantly different according to the LSD test (*p* ≤ 0.05). CorTh is the cortex thickness, XyVZTh is the xylem vessels zone thickness, NoXyV is the number of xylem vessels, DXyV is the diameter of xylem vessels, PiL is the pith length, PiW is the pith width, dI-00 is the irrigation by 100% of crop evapotratnspiration (ETc), dI-20 is the irrigation by 80% of ETc, and dI-40 is the irrigation by 60% of ETc, cP is the conventional soil addition of phosphorus (400 kg P ha^−1^; 15.5% P_2_O_5_), nP-1 is nanophosphorus with 10.8–14.7 nm that was foliarly applied at 0.1 g L^−1^, nP-2 is nanophosphorus with 4.9–8.6 nm that was foliarly applied at 0.1 g L^−1^.

**Table 10:** Leaf anatomical features of fenugreek plants as affected by different irrigation water regimes and soil and foliar nourishing with phosphorus (P) during the season 2019/2020.

Treatments		MidL (µ)	MidW (µ)	VBuL (µ)	VBuW (µ)	NoXyV	LamTh (µ)	PalTiTh (µ)	SpTiTh (µ)
dI-00	cP	625 ^c^	575 ^c^	150 ^c^	200 ^c^	35 ^b^	350 ^c^	200 ^b^	100 ^c^
	nP-1	650 ^b^	600 ^b^	175 ^b^	225 ^b^	40 ^a^	375 ^b^	200 ^b^	125 ^b^
	nP-2	725 ^a^	625 ^a^	200 ^a^	250 ^a^	40 ^a^	400 ^a^	225 ^a^	150 ^a^
dI-20	cP	575 ^d^	525 ^e^	125 ^d^	175 ^d^	30 ^c^	325 ^d^	150 ^d^	75 ^d^
	nP-1	625 ^c^	550 ^d^	150 ^c^	200 ^c^	35 ^b^	325 ^d^	175 ^c^	100 ^c^
	nP-2	650 ^b^	575 ^c^	150 ^c^	225 ^b^	35 ^b^	350 ^c^	200 ^b^	100 ^c^
dI-40	cP	450 ^f^	500 ^f^	100 ^e^	150 ^e^	20 ^e^	275 ^f^	125 ^e^	75 ^d^
	nP-1	550 ^e^	525 ^e^	125 ^d^	150 ^e^	25 ^d^	275 ^f^	125 ^e^	75 ^d^
	nP-2	575 ^d^	550 ^d^	125 ^d^	175 ^d^	25 ^d^	300 ^e^	150 ^d^	100 ^c^
*p*-value		18	15	5	7	2	10	8	5

Means followed by the same letter in each column are not significantly different according to the LSD test (*p* ≤ 0.05). MidL is the midvein length, MidW is the midvein width, VBuL is the vascular bundle length, VBuW is the vascular bundle width, NoXyV is the number of xylem vessels, LamTh is the lamina thickness, PalTiTh is the palisade tissue thickness, SpTiTh is the spongy tissue thickness, dI-00 is the irrigation by 100% of crop evapotranspiration (ETc), dI-20 is the irrigation by 80% of ETc, and dI-40 is the irrigation by 60% of ETc, cP is the conventional soil addition of phosphorus (400 kg P ha^−1^; 15.5% P_2_O_5_), nP-1 is nanophosphorus with 10.8–14.7 nm that was foliarly applied at 0.1 g L^−1^, nP-2 is nanophosphorus with 4.9–8.6 nm that was foliarly applied at 0.1 g L^−1^.

## Data Availability

The data presented in this study are available upon request from the corresponding author.
